# Utility of mean platelet volume in differentiating intrahepatic cholangiocarcinoma from hepatocellular carcinoma

**DOI:** 10.1186/s12876-022-02348-0

**Published:** 2022-06-06

**Authors:** Xin Zhang, Wen-Juan Huang, Meng-Lin Zhang, Wen Wang, Ye Niu, Rui-tao Wang, Zeng-yao Liu

**Affiliations:** 1 Department of Internal Medicine, Harbin Medical University Cancer Hospital, Harbin Medical University, NO. 150 Haping ST, Nangang District, 150081 Harbin, China; 2 Department of Interventional Medicine, The First Affiliated Hospital of Harbin Medical University, NO. 23 Post Street, Nangang District, Harbin, Heilongjiang, 150001 China

**Keywords:** Intrahepatic cholangiocarcinoma, Hepatocellular carcinoma, Mean platelet volume

## Abstract

**Background:**

Intrahepatic cholangiocarcinoma (ICC) and hepatocellular carcinoma (HCC) are the most prevalent histologic types of primary liver cancer. HCC and ICC differ in treatment and prognosis, warranting an effective differential diagnosis between them. This study aimed to explore the clinical value of mean platelet volume (MPV) to discriminate between HCC and ICC.

**Material/methods:**

We performed a retrospective analysis of ICC and HCC patients who were from the Harbin Medical University Cancer Hospital, China. Logistic regression analysis was used to identify the independent factors for the differentiation of HCC and ICC. A receiver operating characteristic curve was built to evaluate the diagnostic performance of the potential model. An independent validation study was performed to validate the diagnostic ability.

**Results:**

ICC patients were detected in 146 out of 348 patients in the primary cohort. MPV levels were decreased in ICC patients compared with those in HCC patients. Logistic regression analysis revealed that MPV was an independent factor in distinguishing HCC from ICC. A combination of sex, hepatitis B surface antigen, MPV, alpha-fetoprotein, and carbohydrate antigen 19–9 demonstrated a good capability to differentiate HCC from ICC. Similar results were achieved in the validation cohort.

**Conclusions:**

MPV may be a new marker to help distinguish ICC from HCC. Further validation studies are required.

**Supplementary Information:**

The online version contains supplementary material available at 10.1186/s12876-022-02348-0.

## Introduction

Primary liver cancer (PLC) is the sixth most common cancer worldwide and the third-leading cause of cancer-related death [1]. The most common types of PLC are hepatocellular carcinoma (HCC) and intrahepatic cholangiocarcinoma (ICC), which account for roughly 95% of all PLC [2, 3]. HCC develops from hepatocytes, whereas ICC develops from biliary epithelium [4, 5]. Although HCC and ICC have overlapping etiological risk factors and clinical manifestations, the therapeutic strategies and prognoses between the two are distinct [2]. Therefore, it is a challenge to differential diagnosis between HCC and ICC.

At present, magnetic resonance imaging (MRI) and contrast-enhanced computerized tomography (CT) are the methods most commonly used to discriminate between the two subtypes, but ICC may mimic the radiological appearance of HCC on CT or MRI and lead to misdiagnosis [6–8]. Approximately 15% of histopathologically confirmed ICC patients who display a “wash-in and wash-out” enhancement pattern on contrast-enhanced CT were misdiagnosed as HCC [7]. Another study revealed that 7–19% of small ICC patients on MRI were misdiagnosed as HCC [9, 10]. Besides, due to the fact that some HCC cases may show the typical enhancement pattern of ICC on contrast-enhanced ultrasound (CEUS), the ability of CEUS to differentiate HCC from ICC is still controversial [11, 12]. Moreover, alpha-fetoprotein (AFP) and carbohydrate antigen 19–9 (CA19-9) are regarded as the blood biomarkers for distinguishing HCC from ICC. However, it is hard to make a distinction between small ICC and HCC in cirrhotic livers due to the low diagnostic sensitivity and specificity of these biomarkers [13, 14]. Elevated AFP is not uncommon in ICC. A series of studies observed that 10.3% of ICC patients had a serum AFP level of > 200 ng/mL [15]. The findings were also confirmed in the study by Zhou [16]. In addition, serum CA19-9 is a frequently used tumor marker for ICC diagnosis but has a low sensitivity and specificity of 53% and 63%, respectively [17, 18]. Thus, there is an urgent need to find new discriminative biomarkers in the clinic.

Platelets' roles in HCC growth have recently piqued the interest of researchers. Mean platelet volume (MPV), an indicator of platelet size, has been proposed as a parameter of platelet function and activation [19]. Furthermore, MPV can serve as a potential biomarker for the diagnosis and prognosis of various tumors, such as lung cancer, thyroid cancer, colorectal cancer, and laryngeal cancer [20–23]. Some reports have proved that MPV is useful as a diagnostic marker for AFP-negative HCC [24, 25]. However, no study to date has clarified the role of MPV in distinguishing ICC from HCC. The objective of this study was to evaluate whether MPV could discriminate between ICC and HCC.

## Materials and methods

### Patients

This study retrospectively reviewed the clinical data of patients histologically diagnosed with ICC or HCC at Harbin Medical University Cancer Hospital, China, between January 2017 and December 2019. Data from another independent cohort of ICC and HCC patients who were diagnosed at the First Affiliated Hospital of Harbin Medical University, from January 2018 to December 2020 was collected retrospectively. Our selection criteria in this study included the following: (1) patients were above 18 years old; (2) pathological diagnosis of HCC or ICC; (3) patients had a curative liver resection. Diagnostic criteria were based on the Guidelines for Diagnosis and Treatment of Primary Liver Cancer in China (2017 Edition). The exclusion criteria included the following: (1) mixed hepatocellular-cholangiocellular carcinoma or other types of liver tumor (n = 36); (2) previous treatment history of HCC or ICC (n = 7); (3) no preoperative AFP or CA19-9 results (n = 6); (4) patients with a history of other cancers (n = 4), diabetes (n = 9), rheumatoid diseases (n = 2), cardiovascular diseases (n = 5), or medical treatment with anticoagulant, statins, or acetylic salicylic acid (n = 7).

The study was approved by the Institutional Ethics Committees of the two hospitals.

### Data collection

All the data was collected from databases. The clinical medical data included demographics, comorbidities, preoperative routine blood tests, biochemistry tests, tumor marker tests, and imaging data. The information on blood tests was obtained from the test report from the Department of Clinical Laboratory. The platelet distribution width (PDW), MPV, and platelet count were directly obtained by an automated hematological analyzer (Sysmex XE-2100, Kobe, Japan).

### Statistical analysis

The statistical analyses were performed using SPSS Statistics version 25.0 (IBM Corp, Armonk, NY). The Kolmogorov–Smirnov test was done to analyze the normally distributed variables. Non-normally distributed variables were expressed as the median and quartile. Logistic regression analysis was used to identify the independent factors for the differentiation of HCC and ICC. Receiver operating characteristic (ROC) analysis was used to determine the potential diagnostic performance of different models in differentiating ICC from HCC. P < 0.05 was considered statistically significant.

## Results

All patients who underwent curative liver resection for ICC or HCC at two affiliated hospitals of Harbin Medical University were enrolled in the derivation set and validation set. The clinicopathological characteristics of patients are summarized in Table 1. Body mass index (BMI), hepatitis B surface antigen (HBsAg), cirrhosis, capsule, nodule diameter, white blood cell count (WBC), haemoglobin, platelet count, MPV, PDW, aspartate transaminase (AST), alanine transaminase (ALT), γ-glutamyl transferase (γ-GGT), total bilirubin, the aspartate aminotransferase/platelet ratio index (APRI), fibrosis-4 (FIB-4), and the neutrophil-to-lymphocyte ratio (NLR) in two groups were significantly different. However, no significant differences were observed between the derivation set and the validation set with regard to age, sex, hepatitis C, tumor number, AFP, and CA19-9 levels. The normal ranges for all the measured variables can be found in Additional file 1: Table S1.

**Table 1:** The characteristics of the patients in derivation set and validation set

Variables	Derivation set	Validation set	*p* value
N	348	158	
Age (years)	55.8 ± 9.5	54.7 ± 10.4	0.246
BMI (kg/m^2^)	24.3 ± 3.3	22.2 ± 2.5	** < 0.001**
Sex (Male, %)	250 (71.8)	102 (64.6)	0.099
HBsAg (%)	197 (56.6)	74 (46.8)	**0.041**
Hepatitis C (%)	19 (5.5)	4 (2.5)	0.143
Cirrhosis (%)	203 (58.3)	66 (41.8)	**0.001**
Tumor number (Multiple, %)	43 (12.4)	23 (14.6)	0.496
Capsule (Incomplete, %)	28 (8.0)	57 (36.1)	** < 0.001**
The largest nodule diameter (cm)	5.4 ± 3.1	6.5 ± 4.6	**0.007**
WBC (× 10^9^/L)	6.04 ± 2.09	8.34 ± 4.09	** < 0.001**
Haemoglobin (g/L)	140.1 ± 15.9	124.7 ± 22.5	** < 0.001**
Platelet count (× 10^9^/L)	182.7 ± 74.8	202.1 ± 99.8	**0.03**
MPV (fL)	14.5 ± 2.5	13.3 ± 2.1	** < 0.001**
PDW (%)	15.2 ± 2.3	13.2 ± 2.2	** < 0.001**
AST (U/L)	29 (22–43)	41 (26–77)	** < 0.001**
ALT (U/L)	30 (19–48)	56 (29–116)	** < 0.001**
γ-GGT (U/L)	65 (33–138)	96 (51–196)	** < 0.001**
Total bilirubin (μmol/L)	14.5 (11.2–18.2)	20.1 (14.3–37.1)	** < 0.001**
AFP (ng/mL)	6.08 (2.61–97.4)	6.23 (2.70–270.1)	0.818
CA19-9 (U/mL)	22.8 (11.3–50.5)	23.5 (12.2–59.4)	0.829
FIB-4	2.8 (1.7–4.7)	1.9 (1.1–3.0)	**0.014**
APRI	0.7 (0.4–1.4)	0.7 (0.3–1.2)	** < 0.001**
NLR	2.87 ± 1.76	4.03 ± 1.94	** < 0.001**

Data are expressed as means (SD) or percentage. SD, standard deviation; BMI, body mass index; HBsAg, hepatitis B surface antigen; WBC, white blood cell count; MPV, mean platelet volume; PDW, platelet distribution width; AST, aspartate transaminase; ALT, alanine transaminase; γ-GGT, γ-glutamyl transferase; AFP, alphafetoprotein; CA19-9, carbohydrate antigen 19–9; APRI, aspartate aminotransferase/platelet ratio index; FIB-4, fibrosis-4; NLR, neutrophil-to-lymphocyte ratio. Bold indicates statistically significant values (P < 0.05)

Table 2 summarizes the characteristics of patients with ICC or HCC. In the derivation set, there were 348 patients, including 202 HCC patients and 146 ICC patients. In the validation set, 158 consecutive patients were studied, consisting of 107 HCC patients and 51 ICC patients. There were more males in the HCC group than in the ICC group. In the derivation cohort, statistical significance was observed in age, sex, HBsAg, hepatitis C, cirrhosis, tumor number, capsule, the largest nodule diameter, APRI, FIB-4, NLR, WBC, haemoglobin, platelet count, MPV, PDW, AST, ALT, γ-GGT, total bilirubin, AFP, and CA19-9 levels between the two groups (Table 2). Other parameters in the two groups were not significantly different.

**Table 2:** The characteristics of the patients with HCC or ICC

Variables	HCC	ICC	*p* value
*Development set*			
N	202	146	
Age (years)	54.3 ± 9.4	57.7 ± 9.3	**0.001**
BMI (kg/m^2^)	24.2 ± 3.0	24.4 ± 3.7	0.487
Sex (Male, %)	163 (80.7)	87 (59.6)	** < 0.001**
HBsAg (%)	157 (77.7)	40 (27.4)	** < 0.001**
Hepatitis C (%)	17 (8.4)	2 (1.4)	**0.004**
Cirrhosis (%)	167 (82.7)	36 (24.5)	** < 0.001**
Tumor number (Multiple, %)	36 (17.8)	7 (4.8)	** < 0.001**
The largest nodule diameter (cm)	4.9 ± 3.3	6.0 ± 2.7	**0.002**
Capsule (Incomplete, %)	11 (5.4)	17 (11.6)	**0.036**
WBC (× 10^9^/L)	5.46 ± 1.92	6.84 ± 2.04	** < 0.001**
Haemoglobin (g/L)	142.5 ± 14.5	136.8 ± 17.2	**0.001**
Platelet count (× 10^9^/L)	166.1 ± 98.5	209.1 ± 79.0	** < 0.001**
MPV (fL)	10.9 ± 1.3	9.9 ± 1.5	** < 0.001**
PDW (%)	14.3 ± 2.5	14.8 ± 2.5	**0.032**
AST (U/L)	33 (24–45)	27 (20–40)	**0.002**
ALT (U/L)	33 (21–49)	26 (17–47)	**0.036**
γ-GGT (U/L)	58 (33–104)	80 (36–185)	**0.002**
Total bilirubin (μmol/L)	15.3 (11.8–20.1)	13.5 (10.2–16.8)	**0.001**
AFP (ng/mL)	19.4 (4.4–425.0)	3.2 (1.87–6.07)	** < 0.001**
CA19-9 (U/mL)	16.4 (9.5–31.1)	44.2 (20.2–470.2)	** < 0.001**
FIB-4	2.1 (1.4–3.2)	1.5 (1.1–2.4)	** < 0.001**
APRI	0.5 (0.3–1.0)	0.4 (0.2–0.6)	** < 0.001**
NLR	1.99 ± 1.36	3.58 ± 1.75	**0.001**
*Validation set*			
N	107	51	
Age (years)	52.7 ± 10.2	58.8 ± 9.6	** < 0.001**
BMI (kg/m^2^)	22.0 ± 2.6	22.7 ± 2.3	0.082
Sex (male, %)	80 (74.8)	22 (43.1)	** < 0.001**
HBsAg (%)	63 (58.9)	11 (21.6)	** < 0.001**
Hepatitis C (%)	1 (0.9)	3 (5.9)	0.064
Cirrhosis (%)	50 (46.7)	16 (31.4)	0.067
Tumor number (Multiple, %)	18 (16.8)	5 (9.8)	0.242
The largest nodule diameter (cm)	6.4 ± 4.2	6.8 ± 5.6	0.636
Capsule (Incomplete, %)	32 (29.9)	25 (49.0)	**0.019**
WBC (× 10^9^/L)	8.14 ± 3.58	8.75 ± 5.00	0.382
Haemoglobin (g/L)	121.8 ± 23.7	130.8 ± 18.7	**0.018**
Platelet count (× 10^9^/L)	178.2 ± 91.5	252.3 ± 98.8	** < 0.001**
MPV (fL)	11.2 ± 1.0	10.3 ± 0.9	** < 0.001**
PDW (%)	13.2 ± 2.2	13.5 ± 2.0	0.431
AST (U/L)	38 (26–61)	55 (25–122)	0.062
ALT (U/L)	50 (30–87)	93 (27–169)	0.058
γ-GGT (U/L)	78 (42–137)	158 (66–413)	** < 0.001**
Total bilirubin (μmol/L)	18.2 (14.0–25.4)	47.0 (16.5–137.1)	** < 0.001**
AFP (ng/mL)	23.7 (4.7–482.4)	2.8 (1.6–3.7)	** < 0.001**
CA19-9 (U/mL)	16.7 (6.8–32.1)	96.6 (28.8–285.7)	** < 0.001**
FIB-4	2.0 (1.1–3.3)	1.6 (1.0–2.8)	** < 0.001**
APRI	0.7 (0.3–1.2)	0.7 (0.3–1.4)	0.132
NLR	3.96 ± 1.76	4.17 ± 1.32	0.548

Data are expressed as means (SD) or percentage. SD, standard deviation; BMI, body mass index; HBsAg, hepatitis B surface antigen; WBC, white blood cell count; MPV, mean platelet volume; PDW, platelet distribution width; AST, aspartate transaminase; ALT, alanine transaminase; γ-GGT, γ-glutamyl transferase; AFP, alphafetoprotein; CA19-9, carbohydrate antigen 19–9; APRI, aspartate aminotransferase/platelet ratio index; FIB-4, fibrosis-4; NLR, neutrophil-to-lymphocyte ratio. Bold indicates statistically significant values (P < 0.05)

Logistic regression analysis was performed to evaluate the risk factors for differentiation of HCC and ICC. In the derivation set, twenty-two variables, including age, sex, HBsAg, hepatitis C, cirrhosis, tumor number, largest nodule diameter, capsule, APRI, FIB-4, NLR, WBC, haemoglobin, platelet count, MPV, PDW, AST, ALT, γ-GGT, total bilirubin, AFP, and CA19-9 entered into the original model. Sex (female vs. male, OR, 3.645, 95%CI, 1.398–9.504, P = 0.008), HBsAg (positive vs. negative, OR, 2.747, 95% CI, 1.054–7.159, P = 0.039), cirrhosis (Yes vs. No, OR, 6.590, 95% CI, 2.648–16.398, P < 0.001), MPV (OR, 1.590, 95% CI, (1.171–2.159), P = 0.003), AST (OR, 1.002, 95% CI, 1.000–1.003, P = 0.018), AFP (OR, 0.974, 95% CI, (0.957–0.991), P = 0.003), and CA19-9 (OR, 1.069, 95% CI, (1.015–1.126), P = 0.011) were the independent risk factors for distinguishing HCC from ICC (Table 3). In the validation set, twelve variables, including age, sex, HBsAg, capsule, haemoglobin, platelet count, MPV, γ-GGT, total bilirubin, AFP, FIB-4, and CA19-9 were entered into the original model. Sex, AFP, CA19-9, haemoglobin, total bilirubin, and MPV were independently associated with the differentiation of HCC and ICC (Table 3).

**Table 3:** Logistic regression analysis of the risk factors for distinguishing HCC from ICC

Variables	β	OR (95% CI)	*p* value
*Development set*			
Sex (Male vs Female)	1.293	3.645 (1.398–9.504)	0.008
HBsAg (Positive vs Negative)	1.010	2.747 (1.054–7.159)	0.039
Cirrhosis (Yes vs No)	1.885	6.590 (2.648–16.398)	< 0.001
MPV (fL)	0.464	1.590 (1.171–2.159)	0.003
AST (U/L)	0.002	1.002 (1.000–1.003)	0.018
AFP (ng/mL)	− 0.026	0.974 (0.957–0.991)	0.003
CA19-9 (U/mL)	0.067	1.069 (1.015–1.126)	0.011
*Validation set*			
Sex (Male vs Female)	2.669	14.424 (2.795–74.439)	0.001
Haemoglobin (g/L)	− 0.058	0.944 (0.910–0.979)	0.002
MPV (fL)	1.388	4.007 (1.226–13.094)	0.022
Total bilirubin (μmol/L)	− 0.014	0.986 (0.973–0.999)	0.040
AFP (ng/mL)	0.007	1.007 (1.002–1.012)	0.008
CA19-9 (U/mL)	− 0.015	0.985 (0.972–0.998)	0.020

OR, odds ratio; CI, confidence interval. Abbreviations: see to Table 1

**Fig. 1:**
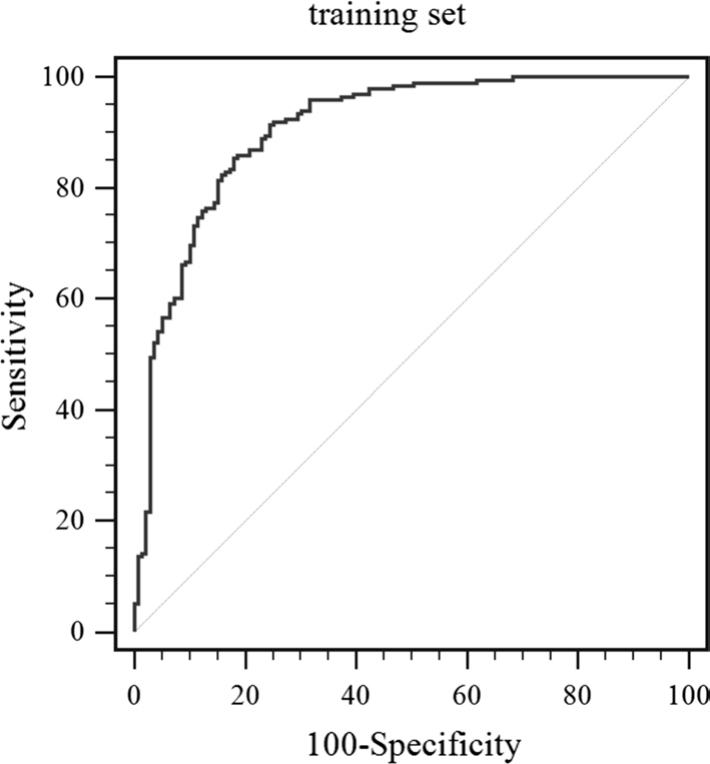
ROC curve for the model showing the sensitivity and specificity of the differential diagnosis of HCC versus ICC in the training set

**Fig. 2:**
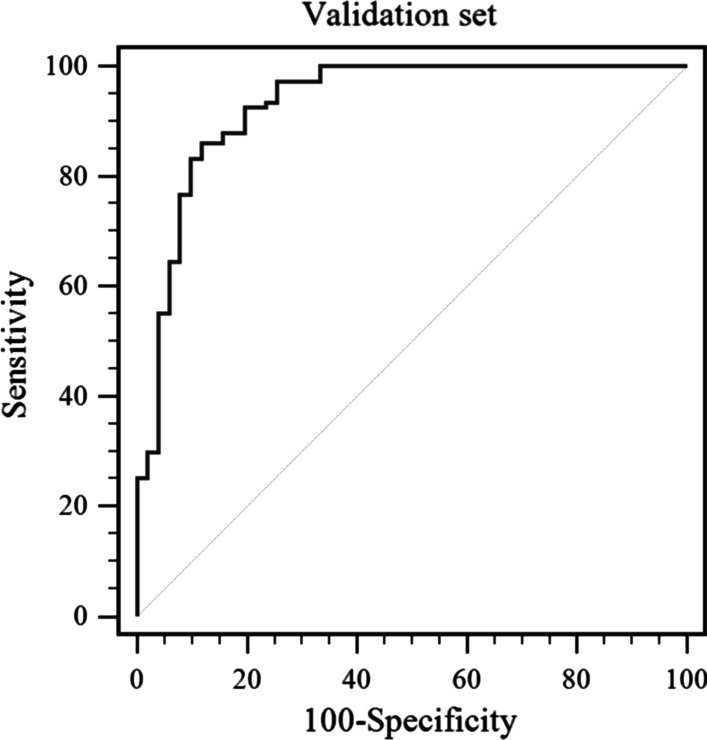
ROC curve for the model showing the sensitivity and specificity of the differential diagnosis of HCC versus ICC in the validation set

**Fig. 3:**
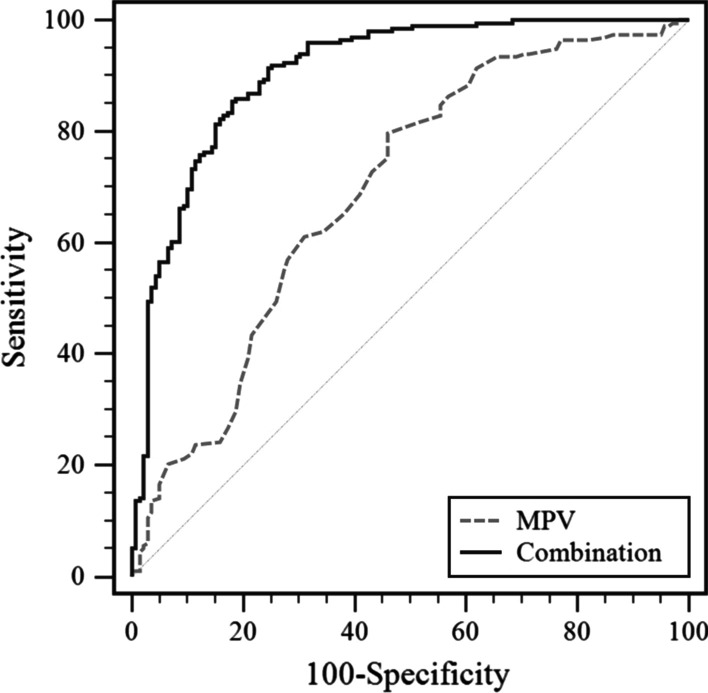
ROC curve analysis showing the capability of the combination of MPV with other variables to differentiate ICC from HCC in the training set

**Fig. 4:**
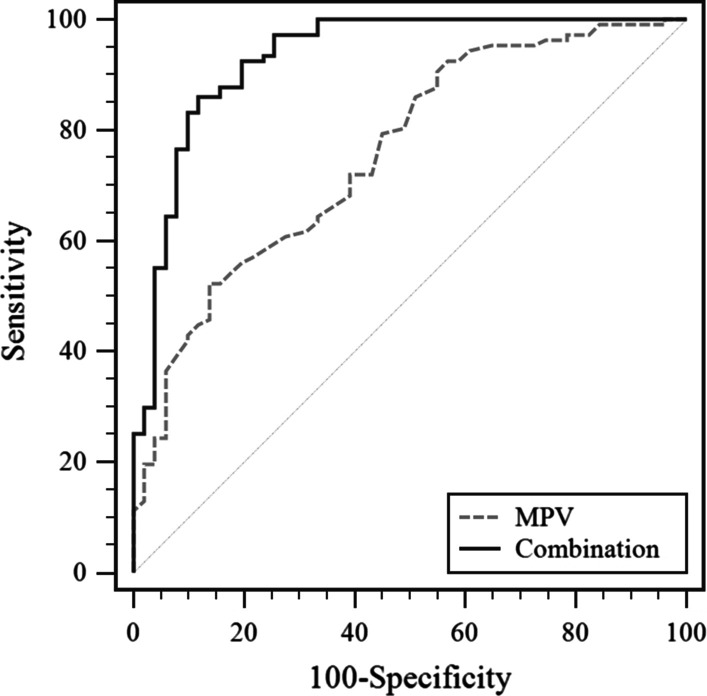
ROC curve analysis showing the capability of the combination of MPV with other variables to differentiate ICC from HCC in the validation set

We built a model incorporating five variables (sex, HBsAg, MPV, AFP, and CA19-9) to discriminate ICC from HCC. The sensitivity, specificity, positive predictive value, and negative predictive value are listed in Table 4. A ROC curve was built to evaluate the diagnostic performance of the potential model. ROC curves showed the sensitivity and specificity of the differential diagnosis of HCC versus ICC in the development set (Fig. 1) and validation set (Fig. 2). For the training set, the model demonstrated a powerful capability to differentiate ICC from HCC, with an area under the curve (AUC) value of 0.907. An independent validation study was performed to validate the diagnostic ability. For the validation set, the C-index was 0.931 (95% CI: 0.880–0.965), demonstrating sufficient accuracy in distinguishing ICC from HCC. The combination of these biomarkers exhibited a significantly larger AUC compared with MPV alone (P < 0.001) (Figs. 3, 4).

**Table 4:** Receiver operating characteristic curve analyses showing the utility of combined markers for differentiating between HCC and ICC

Model	Sensitivity	Specificity	PPV	NPV	AUC
*Development set*					
MPV	0.802	0.541	0.707	0.664	0.698 (0.646–0.746)
Combination	0.854	0.820	0.871	0.797	0.907 (0.870–0.935)
*Validation set*					
MPV	0.863	0.523	0.889	0.463	0.757 (0.683–0.822)
Combination	0.860	0.882	0.939	0.750	0.931 (0.880–0.965)

PPV, positive predictive value; NPV, negative predictive value; AUC, area under curve; Combination, five variables (sex, HBsAg, MPV, AFP, and CA19-9) were included in the model

## Discussion

This study is the first to observe that MPV plays a key role in distinguishing ICC from HCC. Moreover, the external validation cohort came to the same conclusion. These results indicate that MPV might exert distinct functions in the pathology of ICC and HCC.

The gold standard for the diagnosis of PLC is liver biopsy, but for tumors without a biopsy path or with a small diameter, biopsies are usually not available before surgery. Although many markers and techniques have been applied to help clinicians distinguish ICC from HCC [26–28], their clinical value is limited due to the lack of experienced radiologists and costly high-resolution equipment in some developing areas. In our study, we observed that MPV levels in ICC were significantly lower than those in HCC, both in the primary set and in the validation set. Furthermore, MPV may provide additional information to make a distinction between ICC and HCC.

The mechanisms underlying the association of MPV with differentiation are currently unclear. Some reports found that MPV levels in patients with HCC were significantly higher compared to patients with chronic hepatitis or healthy subjects [29]. The authors suggest that MPV could be a potential diagnostic marker for HCC in patients with chronic liver diseases. On the other hand, some studies detected that MPV and MPV-platelet count ratio were significantly higher in HCC patients and were useful for distinguishing AFP-negative HCC patients from healthy individuals [24]. Multiple lines of evidence demonstrate that most HCC develops in an inflammatory environment caused by viral hepatitis and alcoholic or nonalcoholic steatohepatitis [30]. Thrombocytopenia and increased MPV levels in HCC patients may result from decreased activity of thrombopoietin and bone marrow suppression associated with chronic hepatitis C virus (HCV) or hepatitis B virus (HBV) infection and antiviral therapy application [31]. Relative to HCC, substantially less is known about the epidemiology of ICC. Although several ICC-specific risk factors have been identified, such as bile stasis and chronic inflammation of the biliary epithelium, the mechanisms by which they lead to the development of ICC are less clear [32]. Recently, a study revealed that platelets can bind with podoplanin via c-type lectin-like receptor 2 (CLEC-2) and that activated platelets promote liver protection and inhibit liver fibrosis after cholestatic liver injury [33]. Cholangiocarcinoma (CCA) is characterized by a reactive desmoplastic stroma containing enriched cancer-associated fibroblasts (CAFs) that express vascular endothelial growth factor A (VEGF-A) and vascular endothelial growth factor C (VEGF-C), resulting in expansion of the lymphatic vasculature and tumor cell intravasation [34]. Interestingly, previous studies confirmed that platelet-derived growth factor-D increased VEGF-C and VEGF-A production by stimulating CAFs [35].

Although our study observed a new biomarker to distinguish ICC from HCC, some limitations should be taken into consideration when interpreting the findings. Firstly, the heterogeneity of selected patients and statistical bias, such as the overfitting of the model, cannot be fully eliminated. Secondly, because many factors (such as comorbidities, lifestyle, drug usage, and physiology) could influence MPV levels [36], a stricter prospective trial should be designed to confirm the results before the clinical utility of this marker. Lastly, some preanalytical and analytical variables are responsible for the differences in MPV values. The venipuncture procedure, the anticoagulant used for blood collection, the temperature during measurement, different hematological analyzers and measuring methods are the common reasons for the imprecision of MPV evaluation. Standardization of these phases will contribute to a more accurate and reproducible measurement.

## Conclusions

MPV may be a new marker to help distinguish ICC from HCC. Further validation studies are required.

## Supplementary Information

**Additional file 1.** Table S1.

## Data Availability

The datasets used and analyzed during the current study are available from the corresponding author on reasonable request.

## Abbreviations

PLC: Primary liver cancer
ICC: Intrahepatic cholangiocarcinoma
HCC: Hepatocellular carcinoma
MRI: Magnetic resonance imaging
CT: Computerized tomography
CEUS: Contrast-enhanced ultrasound
AFP: Alpha-fetoprotein
CA19-9: Carbohydrate antigen 19–9
MPV: Mean platelet volume
PDW: Platelet distribution width
ROC: Receiver operating characteristic
BMI: Body mass index
HBsAg: Hepatitis B surface antigen
WBC: White blood cell count
AST: Aspartate transaminase
ALT: Alanine transaminase
γ-GGT: γ-Glutamyl transferase
AUC: Area under the curve
HCV: Hepatitis C virus
HBV: Hepatitis B virus
CLEC-2: C-type lectin-like receptor 2
CCA: Cholangiocarcinoma
CAFs: Cancer-associated fibroblasts
VEGF-A: Vascular endothelial growth factor A
VEGF-C: Vascular endothelial growth factor C
PDGF-D: Platelet-derived growth factor-D
SD: Standard deviation
OR: Odds ratio
CI: Confidence interval
PPV: Positive predictive value
NPV: Negative predictive value
APRI: Aspartate aminotransferase/platelet ratio index
FIB-4: Fibrosis-4
NLR: Neutrophil-to-lymphocyte ratio
