# Supplementary material for: Utility of mean platelet volume in differentiating intrahepatic cholangiocarcinoma from hepatocellular carcinoma
Source: BMC Gastroenterol. 2022 Jun 6;22:288. doi: 10.1186/s12876-022-02348-0 (PMC9171941; doi:10.1186/s12876-022-02348-0)
Supplement: Supplementary file 1 — Additional file 1. Table S1. [file 12876_2022_2348_MOESM1_ESM.docx]

**Table S1**

**The normal ranges of all variables measured.**

| Variables | normal ranges |
| --- | --- |
| WBC (×10^9^/L) | 3.5-9.5 |
| Haemoglobin (g/L) | 115-150 |
| Platelet count (×10^9^/L) | 125-350 |
| MPV (fL) | 7-11 |
| PDW (%) | 11-17 |
| AST (U/L) | 13-35 |
| ALT (U/L) | 7-40 |
| γ-GGT (U/L) | 7-45 |
| Total bilirubin (μmol/L) | 3.4-21 |
| AFP (ng/mL) | 0-7 |
| CA19-9 (U/mL) | 0-37 |

Abbreviations: see to Table 1.
